# Sanitary Legislation

**Published:** 1858-04

**Authors:** 


### SANITARY LEGISLATION.

The dissolution of the late Government reduces to symbolical tinder a *Public Health* Bill prepared by Mr. Cowper. As many as seventy large pages are filled with the clauses of this public health proposition, the which, but for the mutability of governmental power, we should now have been discussing with all the weight of argument necessary for so ponderous a document. Suffice it to say over the grave of this unhappy production, that if its author could by his fiat say to the world of progress, "Stop dead short at this present, that my bill may have fair play," the bill, turned into act, might answer some partial purposes; but, as a bill projected at a time when the progress of science is so remarkably rapid, it is the most inconsistent state paper published in modern times. We dispute not that the bill is well intentioned, and drawn up with care; but assert simply, that in the face of the progress stream, it must even have been out of joint in one short year.

*A Letter from the Metropolitan Board of Works to Sir Benjamin Hall* contains a series of objections to the drainage plan proposed by Messrs. Dalton and Simpson, and adverted to in our last. The objections raised are, against open sewers with diminished outfalls delivering at low water; the large increase in the dimensions of sewers to provide for the increased area and rain fall; the enlargement of the area to be drained by gravitation; the use of syphons; and the conveyance of the western sewage to the Surrey side of the river. To this letter, Messrs. Dalton and Simpson have replied with considerable force of argument; and if their plan, as a whole, were commendable, it would not fail from the objections of the Metropolitan Board of Works.

---

### REPORTS AND RETURNS.

*A Report of Mr. Gurney to the first Commissioner of Works on the State of the River Thames* enters into a description of the various kinds of sewage, "the solid, the fluid, and the aeriform", and their distribution. The specific gravity of insoluble sewage being 1·325, it will sink at the rate of a foot per minute in still water, or in slow currents. In currents above 170 feet per minute, the sewage is always in a state of mechanical mixture. The pollution of the Thames is occasioned by precipitation and retention of sewage in the retrogrades and brattice cesspools in the river. To relieve the river of this, Mr. Gurney suggests—

That all the retrogrades and brattice cesspools be destroyed.

That all obstructions to a uniform flow of the river, at low water, be removed.

That the projections along the shore be rounded off, and the hollows filled up.

That the serrated edges of the river at low water be made plain, and continued along the whole line of low water mark.

That the width of the water may, when the tide is at the lowest ebb, be not more than 140 yards from side to side; so that the river may run not only in an uniform current, but at a rate of not less than 225 feet per minute.

The cost of these improvements would be, comparatively speaking, small, and their accomplishment as a temporary sanitary provision, of great public importance.

From a recent *Report on Convict Discipline in Western Australia*, we regret to learn that our friends, the "overfed convicts", are still suffering from the gluttony plague. Dr. Rennie is here again insisting on the reduction of the ration; and Dr. Galbraith, the superior in position, is still in opposition, and is supported in his views by the superintendent, who reports that the frequent change of dietary has caused much discontent among the prisoners, and has given rise to manifestations of feeling of a mutinous tendency.

*A Report on the Spread of Yellow Fever in Bermuda in* 1856 contains much interesting matter, on which we might enlarge freely. Suffice it that Dr. Rees, the Medical Inspector, opines, on sound data, that the disease originated spontaneously in the island.

